# An Unusual Diagnosis of Sporadic Type III Osteogenesis Imperfecta in the First Day of Life

**DOI:** 10.1155/2022/3251980

**Published:** 2022-06-06

**Authors:** Shreeja Shikhrakar, Sujit Kumar Mandal, Pradeep Sharma, Sneha Shrestha, Sanket Bhattarai

**Affiliations:** ^1^Department of Pediatrics, Kathmandu University School of Medical Sciences, Dhulikhel, Nepal; ^2^Kakani Primary Health Center, Nuwakot, Nepal; ^3^Paanchkhal Primary Health Center, Kavre, Nepal

## Abstract

Osteogenesis imperfecta (OI) is a group of rare, permanent genetic bone disorders resulting from the mutations in genes encoding type 1 collagen. It usually is inherited by an autosomal dominant pattern, but it can sometimes occur sporadically. Among the four main types, type III is the most severe type which presents with multiple bone fractures, skeletal deformities, blue sclera, hearing, and dental abnormalities. It is estimated that only 1 in 20,000 cases of OI are detected during infancy, and the diagnosis carries a poor prognosis. This case is reported for the rarity of sporadic OI diagnosis in neonates. We present a case of a 1-day-old neonate following a normal vaginal delivery referred to our center in the view of low birth weight and multiple bony deformities. Physical examination revealed an ill-looking child with poor suckling, gross bony deformities in upper and lower limbs, and blue sclera. X-ray showed thin gracile bones with multiple bone fractures. Echocardiography revealed a 4 mm patent ductus arteriosus. The patient was diagnosed with type III OI with patent ductus arteriosus. Though OI is rare in neonates and infants, it should be considered in the differentials in a newborn presenting with multiple bony deformities regardless of family history, history of trauma, or physical abuse. OI is also associated with cardiac anomalies such as the atrial septal defect and patent ductus arteriosus for which echocardiography is recommended routinely.

## 1. Background

Osteogenesis imperfecta (OI) is a group of rare genetic bone disorders affecting 1 in 13500–15000 births [1]. It results from the mutations in several genes including COL1A1 and COL1A2 genes encoding *α*1 and *α*2 chains of type 1 collagen [1–3]. Around 85%–90% of the cases are inherited by an autosomal dominant pattern and the rest can occur sporadically. Osteogenesis imperfecta (OI) is classified into four types, where type I is the least severe, type II is the lethal type perinatally, type III is the most severe one among survivors, and type IV is of intermediate severity [1, 4]. Type III OI presents with multiple bone fractures, skeletal deformities, blue sclera, hearing, and dental abnormalities [5]. Even though patients with type III OI are thought to have numerous fractures in the fetal and perinatal stages, the findings are often missed in antenatal ultrasonography [2, 5]. Here, we describe a rare case of osteogenesis imperfecta type III with PDA with radiographic findings of diffuse osteopenic changes, multiple fractures, and bone deformities in a newborn. In addition to the rarity of type III OI, only approximately 1 in 20000 cases of OI are detected during infancy [5]. This case is reported for the diagnosis of sporadic type III OI in a 1-day-old newborn.

## 2. Case Presentation

A 1-day-old newborn male born to a 24-year-old primigravida lady was referred to our center with complaints of multiple bony deformities and low birth weight. The baby did not cry immediately after birth and cried only after oronasal suctioning and tactile stimulation. The mother denied a history of trauma or any kind of physical abuse. Mother had regular antenatal visits, but prenatal anomaly scans were not done. There was no significant family history and consanguineous marriage.

**Figure 1:**
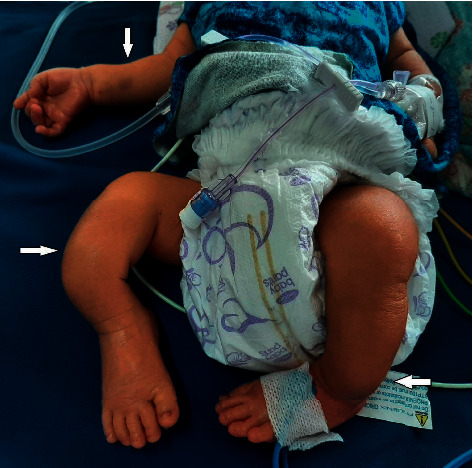
Gross bony deformities present over bilateral upper and lower extremities (shown by arrow head).

On physical examination, the baby was ill-looking, and his vitals were stable. He had a blue sclera but no pallor or icterus. His anterior fontanelle was at level. His tone was normal. He had an ill-sustained and poor suckling. Gross bony deformities were present over bilateral upper and lower extremities (Figure 1). No abnormal findings were noted on systemic examination. He had a low birth weight of 2100 grams and was small for gestational age. His length was 37 cm, which was below the 3rd percentile. The ponderal index was calculated to be 4.

**Figure 2:**
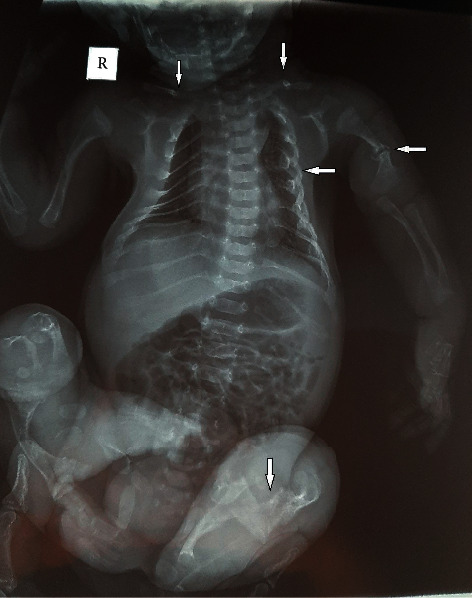
Plain radiograph showing fractures of bilateral clavicles, left humeral diaphysis, bilateral lower limbs, and bilateral ribs which were marked in the left hemithorax (fracture lines shown by arrowhead).

**Figure 3:**
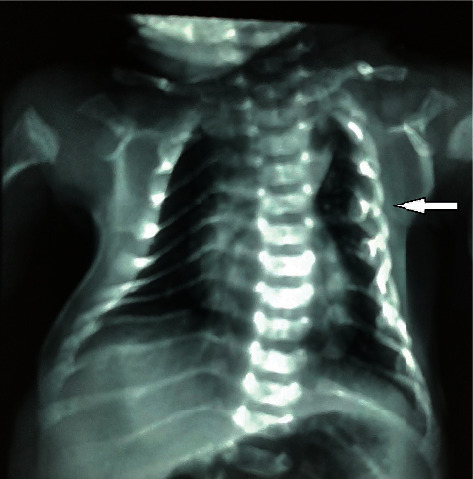
Diffuse osteopenic changes and fractures of ribs (marked more on left hemithorax shown by arrowhead).

Investigations revealed significantly raised white cells (17300 per mm^3^) and differential counts were neutrophil 80 and lymphocytes 12. His platelets were 183000 per mm^3^. His hemoglobin was 15.9 g/dL, and his peripheral blood smear was normal. Serum calcium was 9.1, inorganic phosphorus 7.3 mg/dL, sodium 137 mEq/L, potassium 4.6 mEq/L, urea 30 mg/dL, and creatinine 0.6 mg/dL. He was severely hypoglycemic with random blood sugar less than 20 mg/dL. His total bilirubin was 8.9 mg/dL, and direct bilirubin was 0.8 mg/dL. The X-ray revealed thin gracile bones with multiple fractures including bilateral clavicles, left humeral diaphysis, bilateral lower limbs, and bilateral ribs which were marked in the left hemithorax (Figure 2). Diffuse osteopenic changes and marked bone deformities were seen as incurvation of the bilateral humerus and left radius and ulna suggestive of osteogenesis imperfecta type III (Figure 3). Echocardiography revealed a patent ductus arteriosus (PDA) of about 4 mm.

Based on clinical and radiographic findings, the child was primarily diagnosed as OI type III. The patient was treated with administration of intravenous fluids, ampicillin, and amikacin for 4 days. Oral cholecalciferol 100 IU twice a day and multivitamins once a day were continued along with oxygen supplementation via facemask. On the 1st day of admission, oxygen saturation was maintained in room air and feeding was started. No active intervention was advised, and the patient was discharged on oral cholecalciferol supplementation twice a day to continue along with oral multivitamin once a day to continue. The parents were advised to immunize the child as per Nepal's immunization schedule, provide adequate sun exposure, and breastfeed exclusively for 6 months along with the medications prescribed. At the time of discharge, the child was afebrile, clinically stable, and feeding well. Mother was counseled regarding the proper handling of the baby to minimize fractures and the incurable nature of the disease. She was also advised for a multidisciplinary consultation approach for better management of the condition.

The management was done by a consultant pediatrician with the assistance of pediatric residents at a tertiary care hospital in Nepal. The patient died at the age of 4 months at home which was confirmed via teleconsultation.

## 3. Discussion

Type 1 collagen is the most abundant collagen which consists of two *α*1 chains and one *α*2 chain that are important for maintaining bone strength and is encoded by COL1A1 gene and COL1A2 genes, respectively. Their mutation causes quantitative and qualitative defects of type 1 collagen and noncollagenous matrix proteins resulting in impaired bone strength. In addition, mutations in several genes including IFITM5, SERPINF1, CRTAP, LEPRE1, PPIB, BMP1, SERPINH1, FKBP10, SP7, TMEM38B, SEC24D, CREB3L1, PLOD2, and WNT1 have been implicated in pathogenesis of OI [1, 2]. These genetic defects can be either a quantitative defect presenting with a milder presentation or a qualitative defect presenting with a more severe manifestation [1].

OI is characterized by the triad of fragile bones, blue sclera, and deafness [5]. Owing to the widespread role of type I collagen, the clinical manifestations of OI vary from mild phenotype which is diagnosed later in life to lethal form, which may be diagnosed perinatally [1–3]. The skeletal manifestations also vary with the severity of the disease and include brittle bones, low bone density, multiple fractures, bowing of the extremities, and deformities of the spine which include scoliosis, spondylolisthesis, and vertebral compression fractures. The extraskeletal manifestations may include blue sclera, hydrocephalus, deafness, dentinogenesis imperfecta, dental malocclusion, and pulmonary, cardiac, or gastrointestinal anomalies [1, 3]. Cardiovascular and respiratory manifestations are the leading cause of death in patients with OI. Among the varied cardiac abnormalities such as atrial septal defects (ASD), mitral insufficiency, aortic root dilation, and septal and posterior left ventricular wall thickening, ASD and PDA were the commonest as stated by ElAbd and Moghazy [6].

Furthermore, multiple bone fractures and bone deformities are the predominant skeletal manifestations in OI type III present in the perinatal stages and are fairly uncommon after adolescence [2].

The type III and type II OI can be diagnosed with prenatal ultrasonography revealing bone fractures. In contrast, to type II OI which can be detected as early as 14 weeks of gestation, type III OI is usually detected after 18 weeks of gestation exhibiting severe shortening of the long bones, hypoplastic thorax, marked bowing, fracture, and femur length to abdominal circumference ratio measuring less than 0.16. However, the diagnosis is often missed during prenatal ultrasonography. In a case series reported by John et al. [5], no significant anomaly suggesting OI was seen in 3 out of 3 reported cases in prenatal ultrasonography. These findings can also be confirmed with biochemical analysis such as collagen analysis of skin fibroblast culture or blood deoxyribonucleic acid analysis [5, 7, 8].

Moreover, diagnosis of various forms of OI can also be possible prenatally via gene mutation analysis in the second trimester of pregnancy [9]. The positive family history strongly supports the diagnosis of OI; however, the type of OI may vary in offspring. In addition, the reports of the sporadic occurrence of OI have been reported [2]. In distinction, type IV may occasionally be detected and diagnosis of OI type I is unreliable by prenatal ultrasonography [8].

Since there is no definitive cure to this disease, the management focused on preventing physical disability and improving physical activity [2]. Few management options are available to reduce the manifestations of this disease. There are shreds of evidence suggesting the role of bisphosphonate therapy in increasing the bone mineral density, cortical thickness, and trabecular bone volume and reducing the fracture rate of the long bones in young children with OI [10]. Bisphosphonate therapy is usually initiated after bone density scan [10]. Despite evidences, bisphosphonates could not be initiated in our patient due to various constraints including inaccessibility of the drug amidst COVID-19 pandemic and patient parties' refusal for further treatment in the ground of poor prognosis. Moreover, proper nutrition and activity are the mainstays of treatment of OI including calcium and vitamin D supplementation [3]. In our case, the child was conservatively managed with oral cholecalciferol and adequate sun exposure. Nowadays, bone deformities are treated with surgical osteotomies by drilling small holes in the cortices, and later, minimally invasive nailing is done [2]. However, the child in our case did not undergo any surgery owing to his small age and low birth weight.

The prognosis of OI is seen in terms of patients' ability to walk in later life which is dependent on the type of OI. It has been shown that patients with type III and type IV OI have a lower chance of ultimately walking compared to type I [11]. Babies born with OI type III possess a very bad prognosis. These patients sustain severe disability due to multiple fractures and bone deformities. Since patients with type III OI can have as many as 200 fractures in their lifetime, orthopedic management even with intramedullary nails is difficult. These individuals rarely live past 30 years of age even with proper management [12].

## 4. Conclusion

Though rare, the diagnosis of osteogenesis imperfecta should be kept in the differentials regardless of the family history in a newborn presenting with multiple bony deformities, low birth weight, and low anthropometric measures in the absence of trauma and physical abuse. Prenatal ultrasonography though helpful may not be entirely dependable in diagnosing OI prenatally. Echocardiography should be done routinely in OI for detection of associated cardiac anomalies such as ASD and PDA to increase the survival of the cases.

## Data Availability

The individual medical records used to support this study have not been made available to protect patient privacy. Only those data which do not reveal the patient's identity are available from the corresponding author upon request.
